# Cholera Toxin as a Probe for Membrane Biology

**DOI:** 10.3390/toxins13080543

**Published:** 2021-08-03

**Authors:** Anne K. Kenworthy, Stefanie S. Schmieder, Krishnan Raghunathan, Ajit Tiwari, Ting Wang, Christopher V. Kelly, Wayne I. Lencer

**Affiliations:** 1 Center for Membrane and Cell Physiology and Department of Molecular Physiology and Biological Physics, University of Virginia School of Medicine, Charlottesville, VA 22903, USA; at2ct@virginia.edu (A.T.); tw8zq@virginia.edu (T.W.); 2 Division of Gastroenterology, Boston Children’s Hospital, Boston, MA 02115, USA; stefanie.schmieder@childrens.harvard.edu; 3 Department of Pediatrics, Harvard Medical School, Boston, MA 02115, USA; 4 Harvard Digestive Diseases Center, Boston, MA 02115, USA; 5 Department of Pediatrics, University of Pittsburgh School of Medicine, Pittsburgh, PA 15224, USA; krishnan.raghunathan@gmail.com; 6 Department of Physics and Astronomy, Wayne State University, Detroit, MI 48201, USA

**Keywords:** cholera toxin B-subunit, membrane nanodomains, endocytosis, retrograde trafficking, membrane curvature, membrane rafts, glycolipids, GM1, GL-Lect hypothesis

## Abstract

Cholera toxin B-subunit (CTxB) has emerged as one of the most widely utilized tools in membrane biology and biophysics. CTxB is a homopentameric stable protein that binds tightly to up to five GM1 glycosphingolipids. This provides a robust and tractable model for exploring membrane structure and its dynamics including vesicular trafficking and nanodomain assembly. Here, we review important advances in these fields enabled by use of CTxB and its lipid receptor GM1.

## 1. Introduction

Cholera toxin (CTx) typifies the AB_5_ bacterial toxins, and it is the essential pathogenic factor that causes the massive secretory diarrhea seen in humans infected with *V. cholerae* [1,2]. The Vibrio pathogen first secretes the toxin into the intestinal lumen after colonization of the mucosal surface, but CTx is not active in this space. Remarkably, the toxin encodes within its protein structure everything necessary to breach the intestinal epithelial barrier and enter the cytosol of host cells. Here, in the cytosol, a portion of the toxin induces disease by activation of adenylyl cyclase. This alters the physiology of the intestinal epithelium by activating the Cl^−^ channel CFTR and inhibiting the Na^+^/H^+^ exchanger NHE3 to cause Cl secretion and Na malabsorption leading to a severe form of secretory watery diarrhea [3].

The toxin accomplishes cytosolic entry by co-opting normal aspects of host cell membrane and organelle biology. It does not induce pathogenic membrane pores, or penetrate cell membranes, or damage the integrity of the mucosal surface in any way. Rather, it traffics into the cell and across the mucosal barrier by riding along endogenous pathways of membrane lipid and protein trafficking, and by engaging different aspects of normal sub-cellular organelle biology. The evolutionarily driven adaptations enabling these processes have rendered the toxin one of the most potent and informative probes of cell and membrane structure and function and mucosal tissue biology. This is the topic of the current review: how CTx has illuminated our understanding of basic membrane and subcellular processes fundamental to cell biology.

**Figure 1:**
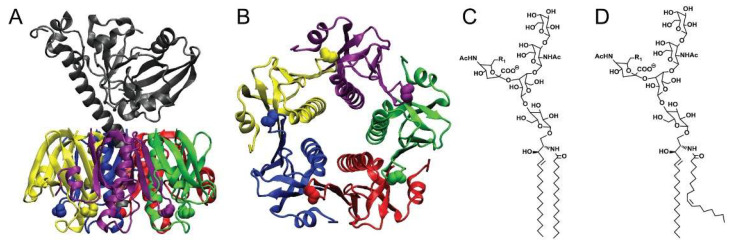
The molecular structures of CTx and GM1. (**A**) A side view of CTx is shown with the A subunit in grey and the five B subunits shown in color. (**B**) A view from the bottom, membrane-binding surface of CTxB. The five G33 amino acids of the GM1 binding pockets are shown as space-filling spheres. (**C**,**D**) Structure of the CTx receptor ganglioside GM1. GM1 structures with ceramides containing acyl chains of C16:0 (**C**) and C16:1 (**D**) are shown. The crystal structure for CTx was downloaded from the Protein Data Bank 1S5E [4]. CTx structure is from O’Neal, C.J.; Amaya, E.I.; Jobling, M.G.; Holmes, R.K.; Hol, W.G. Crystal structures of an intrinsically active cholera toxin mutant yield insight into the toxin activation mechanism. *Biochemistry* **2004**, *43*, 3772–3782. [4].

**Figure 2:**
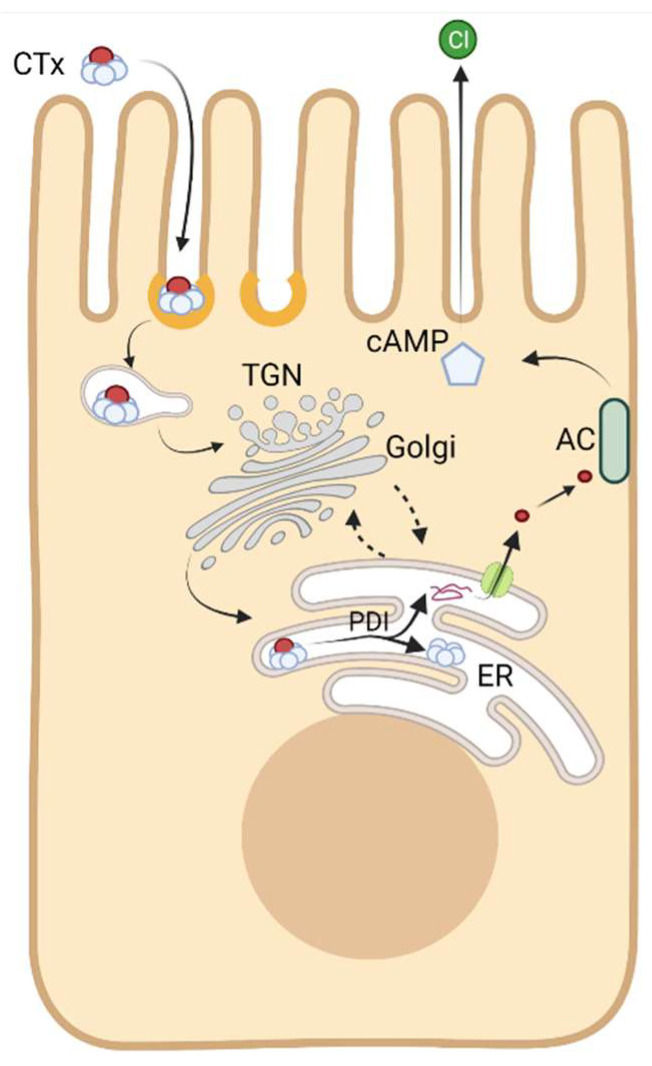
Intracellular itinerary of CTx trafficking and mechanism of intoxification. The CTx holotoxin binds the plasma membrane via its pentameric membrane binding B subunit. It is subsequently internalized and delivered to endosomes. From there the toxin enters into the retrograde trafficking pathway, leading to its delivery to the endoplasmic reticulum (ER). In the lumen of the ER, the A subunit is released from the B subunit and unfolded by protein disulfide isomerase (PDI), enabling its translocation across the ER membrane into the cytoplasm. The A subunit then refolds and ADP ribosylates Gαs. This leads to activation of adenylate cyclase (AC) and increased cAMP levels. Chloride secretion follows, triggering massive watery diarrhea. See text for further details. Created using Biorender.

To enter the cytosol of host cells, CTx has evolved to bind with high affinity to the oligosaccharide moiety of a raft-associated glycosphingolipid, ganglioside GM1. This is mediated entirely by the toxin’s binding B-subunit, CTxB (Figure 1 [4]). CTxB assembles as a homopentamer of 11 kDa peptide chains and it functions as a lectin with five binding sites for the oligosaccharide head group of GM1 (and other closely related gangliosides [5,6,7]). It is thus capable of clustering up to five glycosphingolipids together [8]. GM1 acts as the vehicle for endocytic uptake and retrograde trafficking of CTx all the way backwards in the secretory pathway into the endoplasmic reticulum (ER) [9,10,11]. The structure of the ceramide moiety of GM1 dictates the trafficking of the toxin-GSL complexes in this pathway [12,13]. Once in the ER, the CTx A-subunit co-opts the mechanics of protein folding quality control to separate from the B subunit and retrotranslocate across the ER-limiting membrane into the cytosol where it induces toxicity by enzymatically ADP-ribosylating Gαs and activating adenylyl cyclase (Figure 2) [14,15]. Separate secondary and lower affinity binding sites for glycoproteins also exist on CTxB that modify toxin action [16,17,18,19,20,21,22,23,24,25,26,27].

Here, we focus in this review on how the CTxB subunit can be used as a non-toxic reporter to probe basic aspects of membrane structure, mechanisms of endocytosis, nanodomain assembly, and membrane trafficking enabled by glycosphingolipid biology.

## 2. CTxB as a Probe for Membrane Organization

**Figure 3:**
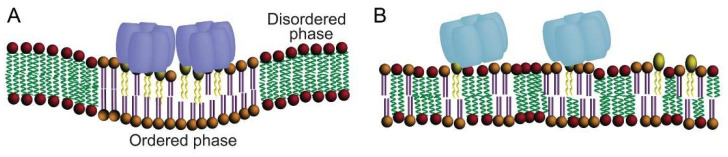
Multivalent binding of CTxB binding to order-preferring GM1 is required to induce phase separation and stabilize ordered membrane domains. (**A**) Wild type CTxB can bind to up to 5 GM1s. As a consequence of multivalent binding to GM1, CTxB can generate and sort to order-preferring lipid phases. Multivalent binding also induces membrane curvature (see Figure 4 for further details). (**B**) A CTxB mutant containing a single binding functional GM1 site associates equally well with ordered and disordered domains. It is also incapable of stabilizing raft domains or inducing membrane curvature. Wild type CTxB is predicted to behave similarly under conditions where it binds to a single GM1, for example at low GM1: CTxB ratios. Note that for simplicity, cholesterol is not depicted in the membrane.

The plasma membrane is thought to contain over 100,000 different lipid species whose distributions within the membrane leaflets are not homogeneous. Instead, these lipids, along with membrane-associated proteins are often organized laterally into domains based on differential physicochemical interactions [28]. One well-studied example of membrane organization is membrane (lipid) rafts, which are regions enriched in sterols, sphingolipids and saturated phospholipids [29,30]. Rafts are defined by having altered membrane miscibility, highly packed and tightly ordered lipid molecules characteristic of lipids in the liquid ordered (Lo) phase, distinct from a more fluid liquid disordered (Ld) membrane environment [31]. The biology of CTx is closely linked to membrane rafts [32,33,34,35], and over the last two decades, CTxB has often been used as a marker for rafts and thereby for deciphering their properties and physiological functions [29,36,37]. In this section, we focus on several recent studies seeking to uncover the mechanisms that control the association of CTxB with rafts and related membrane nanodomains (Figure 3). Membrane rafts are thought to range in size from few to a couple of hundreds of nanometers. Likewise, their lifetimes are also thought to vary from nanoseconds to much longer times under conditions where they are stabilized [29]. Given the inherent difficulty of experimentally elucidating such dynamic and diffraction-limited processes as well as their composition in cells, many of the characteristics of lipid rafts in cells are still under debate [38,39,40,41]. Several fundamental properties of rafts can, however, be studied in model systems such as giant unilamellar vesicles (GUVs) and giant plasma membrane vesicles (GPMVs) [42,43]. Depending on the lipid composition and experimental conditions, membranes in GUVs and GPMVs form co-existing L_o_ and L_d_ domains.

CTxB associates with the ordered phase of GUVs containing trace amounts of GM1, but this depends on the structure of its ceramide moiety (see further discussion below) (Figure 1C,D) [44]. CTxB also can induce domain formation in single-phase GUVs comprised of lipids close to a demixing point, suggesting that the toxin can actively reorganize the membrane to form domains [45,46]. Typically, CTxB preferentially associates with and stabilizes ordered raft-like domains in GPMVs derived from living cell plasma membranes [12,44,45,47,48,49,50,51,52,53,54]. These findings led to the hypothesis that CTxB assembles stabilized raft domains via its ability to cluster together multiple copies of GM1. We recently tested this idea in GPMVs using a monovalent variant of CTxB capable of only binding to a single GM1 [55]. Consistent with the predictions of this hypothesis, a monovalent mutant CTxB did not bind preferentially to Lo nanodomains—rather it bound equally well to the ordered and disordered phases [55]. Since GPMVs retain the same lipid complexity as biological membranes [54], similar stabilization of rafts likely occurs upon binding of CTxB to the plasma membrane of living cells.

The intrinsic preference of GM1 itself for raft or non-raft domains also influences the phase in which CTxB preferentially resides. GM1 is classically thought to be a raft-associated glycolipid [29,36,43,45]. However, some GM1 species with unsaturated acyl chain lengths do not associate with raft domains. We recently investigated how these key structural features of GM1 influence its phase preference [13,56]. Headgroup-labeled fluorescent GM1 species containing a C16:0 acyl chain partition into the Lo phase, whereas GM1 with a C16:1 acyl chain do not, even when clustered by CTxB [56]. A small library of GM1 species was also recently tested for Lo and Ld phase preference in GPMVs obtained from various cell types. Here, we found that the partitioning between phases depended on the presence or absence of unsaturated cis-double bonds in the acyl chain of the ceramide moiety [13]. Normally in cells, the most prominent acyl chain structures include palmitic, stearic and nervonic acid [57]. How CTxB would behave when bound to multiple but different GM1 species containing different combinations of acyl chains remains to be investigated.

GM1 is also known to form nanoclusters in cell membranes and model membranes [58,59,60]. There is wide discrepancy in the reported size, composition and phase of these clusters in model systems [60]. In cells, GM1 nanoclusters have been reported to form in a cholesterol, actin, and temperature-dependent manner [58,59]. Our recent work has revealed that the ability of GM1 species to form nanoclusters is also controlled by their ceramide structure [56]. As reported by fluorescence anisotropy homoFRET measurements, GM1 containing a C16:0 acyl chain forms nanoclusters in live cell membranes. These nanoclusters are cholesterol, phosphatidylserine, and actin-dependent, suggesting that they share some features previously reported for nanoclusters of GPI-anchored proteins [61]. In contrast, GM1 with a C16:1 acyl chain is predominantly randomly distributed across the cell surface [56]. The addition of CTxB induces higher order clustering within live plasma membranes, leading to the formation of domains which are stable over a timescale of seconds [56]. While an increase in cluster size was observed for both saturated and unsaturated GM1 upon CTxB binding, the nanodomain properties differed depending on GM1′s acyl chain. The function of the GM1 nanodomains is not yet clear, but could represent sites where CTxB initially binds the membrane or sites that dictate lipid sorting through the different recycling, retrograde, and late endosome/lysosome endocytic pathways [13]. They may for example be linked to transport of CTx from the plasma membrane to the Golgi complex, a process already known to be actin dependent [62].

Taken together, these findings emphasize the importance of the structure of both CTxB and GM1 in controlling their association with rafts and cellular nanodomains. This has broader implications for our understanding of how AB_5_ toxins regulate their association with membrane domains, as well as the general roles that lipid acyl chain structure and protein-mediated lipid clustering events play in membrane organization. They also raise interesting questions about how the structure of CTxB and GM1 controls additional biological activities of the toxin such as its ability to sense and/or induce curvature in cell membranes and influences its endocytic and intracellular trafficking, as discussed further below.

## 3. CTxB as a Sensor and Inducer of Membrane Curvature

Regulated membrane shapes are critical to diverse cellular processes such as exocytosis/endocytosis, pathogen vulnerability/protection, therapeutic targeting, and organelle morphology [63]. Proteins have shown a diverse capability to sense and generate curvature by a variety of mechanisms depending on both the membrane and protein properties [64,65,66,67]. CTxB has emerged as an important model and tool to understand how proteins affect and are affected by membrane curvature. CTxB exhibits an intrinsic capability to manipulate membrane shapes, as shown by CTxB-induced membrane budding in quasi-one component model lipid bilayers [68,69]. In cells, CTxB localizes to the inside of membrane tubules and vesicles as it is trafficked from the plasma membrane to the ER. Current models suggest that CTxB and related toxin B-subunits such as that of Shiga toxin (STxB) not only prefer to reside in regions of negative membrane curvature like those found inside transport carriers but also induce de novo curvature upon binding to membranes via a cooperative process [36,68,70,71,72,73,74,75,76,77,78]. Thus, curvature sensing and generation are key to the toxin’s biological activities.

To provide mechanistic insights into how CTxB induces membrane curvature, we recently employed polarized localization microscopy (PLM), a form of super-resolution microscopy that detects membrane curvature [69,79]. PLM combines single-molecule localization microscopy with polarized total internal reflection microscopy to reveal membrane curvature. By varying the ratio of GM1: CTxB in model membranes of controlled composition, we found that CTxB requires a stoichiometry of binding with at least two GM1 molecules per CTxB to generate curvature [79,80]. This was confirmed by comparing pentavalent wild type (wt) or a monovalent mutant CTxB (mCTxB) capable of binding only a single GM1 in both cells and model membranes [79]. In all conditions, multivalent binding was critical for CTxB to induce membrane shape changes.

**Figure 4:**
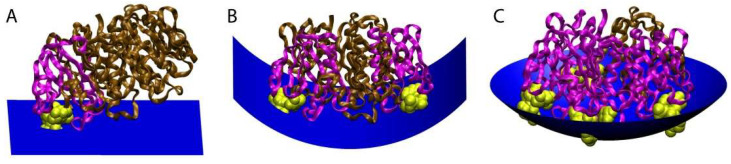
Model for how the stoichiometry of binding of CTxB to GM1 controls the degree of membrane curvature. The ratio of CTxB subunits bound (pink) and unbound (brown) to GM1 (yellow) affects the shape of the membrane surface (blue). (**A**) Binding of CTxB to a single GM1 has no effect on membrane curvature. (**B**) Binding to two GM1s generates cylindrical negative curvature in one dimension. (**C**) Binding of CTxB to three or more GM1s induces spherical negative membrane curvature in two dimensions. This results in a wrapping of the membrane around the CTxB, such as within an endocytic pit. The schematics were created in Visual Molecular Dynamics [84] by building upon a crystal structure of CTxB [85]. Figure reproduced with permission Kabbani, A.M., Raghunathan, K., Lencer, W.I., Kenworthy, A.K., and Kelly, C.V. Structured clustering of the glycosphingolipid GM1 is required for membrane curvature induced by cholera toxin. *Proc. Natl. Acad. Sci. USA* **2020**, *117*, 14978–14986 [79].

CTxB likely induces membrane curvature by several mechanisms, all of which depend on its ability to cluster multiple GM1s. The first is a direct consequence of the physical shape of CTxB and relative locations of the five GM1 binding pockets. In particular, the GM1 binding pockets on CTxB are located on the perimeter of the homopentamer and elevated above the membrane-binding surface of CTxB. Because of this the membrane must deform to enable binding to more than one GM1 simultaneously (Figure 4). According to this model, upon engagement of the first GM1 CTxB assumes a tilted orientation, minimally perturbing membrane shape [72,81,82,83]. In response to binding of CTxB to two or more GM1s, however, the membrane bends to allow GM1 to reach the peripheral GM1 binding pockets on the CTxB. Multivalent binding of GM1 to CTxB ultimately requires the membrane to wrap around the CTxB [72,77]. The degree of induced curvature is thus directly linked to the GM1:CTxB stoichiometry.

The ability of CTxB to induce membrane phase separation in response to GM1 crosslinking also contributes to its membrane bending activity. Local enrichment and crosslinking of GM1 by CTxB creates a local membrane composition enriched in order-preferring gangliosides, which may trigger lipid phase separation. Lipid phase separation can in turn encourage membrane bending by creating a line tension or differential lateral pressure profiles [86,87,88,89]. Coupling between the lipid phase and membrane curvature would thus be expected to initiate a cooperative feedback loop that amplifies membrane curvature [90].

Crosslinking of GM1 by CTxB may also induce local lipid compression and membrane tension changes, similar to that observed upon crosslinking of Gb3 by STxB [78]. This is important because membrane tension is key to membrane shape regulation [67]. In model membranes consisting primarily of POPC, CTxB induces similar membrane curvature when bound to GM1s that differ in the length and saturation of their acyl chains [79]. This implies that curvature generation is independent of the acyl chain composition of GM1 and that lipid phase separation is not required for CTxB to induce membrane shape changes [79]. Since both CTxB and STxB induce negative membrane curvature and are localized to the outer leaflet of the plasma membrane, it is unlikely that toxin crowding generates steric pressure since this would be counterproductive to endocytosis [91]. Instead, toxin binding may induce asymmetric membrane tension via compression of glycolipids in the outer leaflet to foster the negative membrane curvature. These local tension variations across the plasma membrane may in turn facilitate toxin internalization [92].

CTxB molecules must also act cooperatively to facilitate large-scale membrane shape changes [93]. The multivalent binding of CTxB to GM1 may also play an important role by facilitating the formation of local membrane hotspots of lipid phase separation and curvature [69,94]. One potential mechanism that could contribute to the local accumulation of CTxB is membrane-mediated attractive forces extending >20 nm between CTxBs [93,95,96]. These forces would be expected to be further amplified by membrane shape undulations [97,98]. The collective action of multiple CTxBs to bend cell membranes likely employs a three-dimensional scaffolding to generate membrane topographies orders-of-magnitude larger than a single CTxB [99].

In summary, CTxB induces and senses membrane curvature through a complex interplay of physical factors resulting from its molecular shape and lipid crosslinking. Recent results have demonstrated that CTxB locally accumulates and induces membrane bending though the collective result of lipid phase separation, compression, and crosslinking inherent in the multivalent binding of GM1 by CTxB. Ongoing efforts will focus on the precise mechanisms by which local physical changes to the membrane composition and shape recruit the downstream endocytic and intracellular trafficking machinery, for which WT and mutant CTxB are likely to be key tools for research.

## 4. CTxB as a Reporter of Clathrin-Independent Endocytosis

Endocytosis- a processes by which a plethora of molecules such as nutrients, extracellular cargoes, and activated membrane proteins/receptors are internalized into the cell- is critical for physiological proceedings such as nutrient uptake and intracellular signaling driving cellular homeostasis (reviewed in [100,101,102,103]). Endocytosis also drives cellular infection induced by bacterial and viral proteins and/or toxins (selected reviews include [104,105,106,107]). The process of endocytosis occurs at plasma membrane. Initiation of endocytic events induces biophysical modifications of the plasma membrane, leading to membrane invagination and scission of endocytic carriers to facilitate the uptake of molecules/cargoes into the interior of the cell.

Mechanistically, endocytosis can be broadly classified into clathrin-dependent and clathrin-independent pathways (extensively reviewed in [108,109,110]). Driven predominantly by the presence and activity of clathrin, clathrin mediated endocytosis (CME) is well-studied and characterized at molecular level [111,112,113]. In contrast to CME, the molecular determinants and mechanisms of clathrin independent endocytosis (CIE) differ depending upon the morphology of the endocytic carriers, cargoes and physiological need of the cells (reviewed extensively in [114,115,116,117,118,119,120,121,122]). Furthermore, a unique or a universal molecular player for CIE is yet to be defined and our knowledge about CIE machinery is continuing to evolve.

Studies on the uptake of cholera toxin and in particular its B subunit CTxB have not only enhanced our understanding of the endocytic itinerary and pathophysiology induced by bacterial toxins but also enlightened us about the biophysical modifications that the plasma membrane undergoes in preparation for endocytosis. In this section, we will briefly discuss about different routes of clathrin independent internalization of CTxB. Further, we will also touch upon the utility of CTxB in delineating bacterial toxin internalization pathways that in turn has broadened our knowledge about modalities and machineries driving CIE pathway in general.

**Figure 5:**
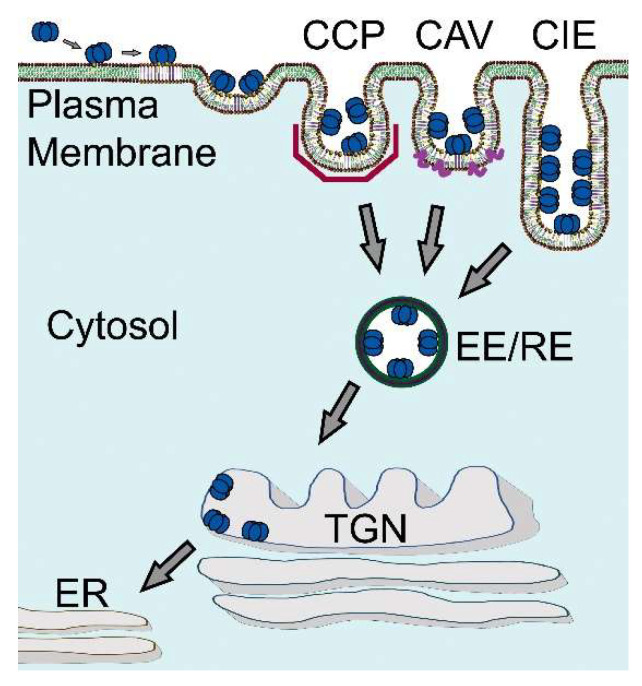
Overview of the intracellular trafficking of CTxB. The biological function of CTxB is to carry the enzymatically active A subunit of cholera toxin into cells. To do so, CTxB must first bind GM1 on the host cell membrane. The subsequent entry of CTxB into cells depends importantly on its ability to cluster multiple GM1s. This enables CTxB to induce and/or sort into areas of negative membrane curvature and to enter cells via raft-dependent, clathrin-independent endocytic pathways (CIE). It can also be internalized via clathrin coated pits (CCP) and caveolae (CAV). Following endocytosis, CTxB is trafficked to endosomal compartments such as early endosomes (EE) and recycling endosomes (RE). To induce cellular intoxification, CTxB must undergo additional retrograde trafficking steps to the trans-Golgi network (TGN) and endoplasmic reticulum (ER). Which intracellular trafficking pathways CTxB ultimately follows is controlled in part by the structure of the ceramide moiety of its receptor GM1.

Early on, it was recognized that CTxB can enter cells via multiple mechanisms, including both canonical clathrin-dependent endocytosis and clathrin-independent mechanisms [123,124,125] (Figure 5). For example, CTxB can be internalized via flask-shaped plasma membrane invaginations known as caveolae [126,127,128]. It is also taken up into cells via the CLathrin-Independent Carriers (CLICs) and GPI-Enriched Endocytic Compartments (GEECs) pathway, a dynamin-independent endocytic pathway responsible for the uptake of a variety of raft-associated proteins including GPI-anchored proteins [125,129,130,131,132,133]. Another dynamin- and caveolin-independent CIE mechanism utilized by CTxB is Arf6-mediated endocytosis, a pathway that internalizes similar cargoes as the CLIC/GEEC pathway [121,124,134,135].

More recent work has revealed that cholera toxin activates and is internalized by a clathrin-independent pathway dubbed fast-endophilin mediated endocytosis or FEME [116,118,136,137,138,139]. Driven by the N-BAR domain protein endophilin A2 (endoA2), the FEME pathway is activated at the leading edge of migrating cells in response to ligand binding to receptor tyrosine kinases and G-coupled receptors (GPCR), which are subsequently taken up by the pathway [137,138,139]. Endophilin plays several important roles in this pathway, including capture of transmembrane receptor cargo, generation of membrane curvature, and scission of tubular endocytic carriers in cooperation with dynamin and actin via a friction driven process [136,137,140]. Microtubules and dynein play important roles in the FEME pathway as well, contributing to membrane tubulation and scission [136,141,142]. Interestingly, CTxB is capable of activating FEME: in response to CTxB binding, endoA2 is recruited to the plasma membrane, resulting in uptake of CTxB into endoA2-positive carriers. Internalization of CTxB is reduced upon knock down of EndoA2, further implicating FEME as a mechanism that controls toxin uptake [136,143]. Additional machinery that regulates the FEME pathway is continuing to emerge [142,143].

An important question raised by these findings is how toxin binding is sensed by the cell and translated into a signal that triggers endocytosis. One hypothesis is that clustering of multiple copies of GM1 upon CTxB itself serves as a signal. According to this model, the ability of the toxin to bind multiple copies of GM1, as well as structured clustering of GM1 by CTxB, functionally regulate toxin internalization. In support of this idea, toxin variants engineered to contain as few as one GM1 binding site exhibit strongly attenuated internalization, although they are still capable of completing the intoxification pathway [144,145]. One major consequence of multivalent glycolipid binding, as discussed above, is induction of membrane curvature [79]. This principle was first identified for the case of STxB and represents an example of a broader mechanism whereby lectins generate membrane curvature to drive endocytosis by binding to multiple glycolipids or glycoproteins (the GL-Lect hypothesis) [146]. Membrane curvature created by the extracellular CTxB could potentially lead to the recruitment of intracellular curvature-sensing proteins, in turn controlling the local membrane composition [147,148]. However, CTxB mutants capable of binding to only a single copy of GM1 can sort into preformed clathrin-independent endocytic structures, suggesting glycolipid clustering-induced curvature generation is dispensable for its uptake into at least a subset of CIE carriers [141].

As discussed above, the binding of CTxB to multiple copies of GM1 also regulates its association with ordered domains and the ability of the toxin to stabilize raft domains [55]. Glycolipid crosslinking initiated by cell surface binding of cholera toxin and other AB_5_ family toxins such as STx also initiates signaling events [149]. For example, binding of STx upregulates the formation of clathrin-coated pits, and modulates microtubule dynamics [150,151]. It seems likely that these raft-stabilizing activities and signaling capacities of CTxB, combined with local curvature generation that drives the recruitment of the intracellular endocytic machinery, contribute to its endocytic uptake by generating a “curvature-signaling hub”. However, the exact mechanisms by which these processes are coupled remain to be determined.

In conclusion, internalization of CTxB occurs via multiple mechanisms and depends on a variety of molecular players operating at the plasma membrane on different time scales. CTxB actively regulates several of these pathways by inducing changes in membrane organization and intracellular signaling in response to toxin binding. These activities are enhanced by CTxB’s ability to cluster multiple GM1s, but glycolipid clustering is not essential for internalization of CTxB or the ability of CTx to cause cellular intoxification. However, many open questions remain about how these events are orchestrated. It is thus clear that CTxB will continue to be a critically important tool to advance our knowledge of bacterial toxin internalization as well as to further illuminate our understanding of mechanisms of CIE.

## 5. CTxB as a Probe of Retrograde Trafficking Mechanisms

The ultimate destination of internalized CTxB is the ER. The endocytic network of all cells includes a pathway from the plasma membrane retrograde to the trans-Golgi network (TGN)—and for the glycosphingolipids all the way backwards in the secretory pathway to the ER [152]. For example, the pathway operates to regulate recycling of the mannose-6-phosphate receptor, the endosome protease furin, and the endogenous glycosphingolipids. Trafficking of CTxB and the other enteric AB_5_ toxins from cell surface all the way into the ER requires binding to membrane glycosphingolipids of the host cell, followed by endocytosis, endosomal sorting, and transport of the CTxB-GM1 complex into the retrograde pathway (Figure 5) [153,154]. The retrograde pathway links the cell surface with the trans-Golgi complex and the ER by vesicular trafficking. This pathway was first discovered in studies on STx by Sandvig and van Deurs using thin-section electron microscopy [155]. This marked a fundamental turning point in our understanding of how these toxins entered host cells to cause disease.

STx, CTx and other AB_5_-toxins have since provided robust tools to study the mechanisms and components responsible for endosomal sorting and membrane trafficking in the retrograde pathway (summarized before in [11,77,152]). Toxin trafficking was measured by immune or direct labeling of the toxins with fluorophores or nano-gold particles or biochemically by tagging the toxins with N-glycosylation motifs that became glycosylated when the toxins entered the ER lumen [33,156]. These studies were highly informative, but none were able to measure retrograde trafficking in real time or quantitatively. In addition, both the imaging and biochemical approaches were technically demanding, which prevented their application to high content and high throughput unbiased genetic or chemical screens.

To address these problems, we modified the new split fluorescent protein technologies to link a small fragment of GFP to CTx (via fusion to the A2-chain, termed CTB-mNG2_11_) [10,157]. The approach led to the development of a novel quantitative and near real-time single-cell flow cytometry assay for retrograde membrane transport driven by CTxB binding to GM1 [10]. Retrograde trafficking to either the TGN or ER was monitored in cells stably expressing the GFP acceptor fragment (mNG2_1–10_ GFP) fused to TGN or ER targeting sequence by quantifying the evolution of a fluorescence signal upon binding of CTB-mNG2_11_ and mNG2_1–10_ GFP. The assay led to the discovery that perturbations of the sheet and tubular morphology of the ER affects the retrograde trafficking pathway. Moving forward, this approach should be fully amenable to high throughput studies on the underlying biology of membrane trafficking.

In a second approach, we have directly visualized the trafficking of GM1 itself through the use of fluorescent headgroup-labeled forms of GM1 [12,13,158]. These lipids enabled us to monitor retrograde trafficking of GM1 in the presence and absence of bound CTxB and dissect the role of ceramide structure in dictating the trafficking pathways utilized by GM1. These are important questions given that lipids themselves are sorted in endosomal pathways [159] and reports that trafficking of other AB_5_ toxins are affected by the structure of their glycolipid receptors [160]. We discovered that retrograde sorting of the CTxB-GM1 complex depends importantly on the structure of the GM1 ceramide moiety [12,13]. Ceramide structure, driven by the length of the ceramide acyl chain and position of any cis double bonds if present [13], enables sorting of the toxin (and the glycosphingolipid itself) into the narrow and highly curved sorting tubules emerging from the early sorting endosome compartment. These sorting tubules feed the recycling, retrograde, and (in polarized cells) transcytotic pathways. Fully saturated ceramide acyl chains drive the lipid away from sorting tubules causing retention of GM1 in the body of the sorting endosome and maturation into late endosomes and lysosomes. Notably, sorting of the different GM1 species among these pathways correlates with the ability of the different ceramide structures to regulate the association with cholesterol-dependent membrane nanodomains [13]. As GM1 acts as the trafficking receptor for the 84 kDa CTx, we believe these discoveries might be harnessed for clinical applications that require transport of therapeutic peptides and proteins into the various sub-cellular compartments of cells and even across mucosal barriers by transcytosis [158,161,162,163,164].

Altogether, these recent advances highlight the continued importance of CTx and GM1 as reporters of retrograde trafficking pathways. The tools described here should ultimately help develop a deeper understanding of mechanisms controlling the intracellular transport of CTx and related toxins, uncover principles that govern both protein and lipid sorting at multiple sites within cells, and design new strategies for delivery of therapeutics.

## 6. Alternative Membrane Glycoprotein Receptors Affecting CTx Biology

CTxB binds the ganglioside GM1 with high affinity, and GM1 has been shown with the greatest clarity to act as the functional receptor leading to CTx entry into the ER of host cells and the induction of toxicity [12,13,144,165,166,167,168,169,170,171,172]. However, it has long been known that CTx will also bind other glycosphingolipids [7,167,173,174,175,176,177,178,179,180,181]—and even glycosylated proteins, including the histo-blood group antigens [24,25]. The site of (low affinity) binding to the histo-blood group antigens was recently elucidated and found to occur on the side of the B-pentamer separate from the site where the B-subunit binds the glycosphingolipids [26]. Some reports show evidence consistent with the idea that these glycoproteins can act on their own, like the glycosphingolipids, to enable endocytic uptake and retrograde trafficking of CTx into the ER required for the induction of toxicity [27].

The evidence for secondary receptors, though in many ways compelling, is largely circumstantial and could have alternative explanations. The glycoprotein receptors do lead to endocytic uptake, for example [182]. However, for our part, we do not think binding to the glycoproteins function in the retrograde pathway. First, none of the implicated glycoproteins have been shown to traffic retrograde all the way into the ER; this is a very unusual pathway for plasma membrane proteins. Additionally, more unambiguously, point mutations in the primary binding site for the glycosphingolipids fully inactivate toxicity [85,168]. Thus, binding to the glycosphingolipids is essential. The secondary glycoprotein receptors for CTx do, however, modify toxin action, and the histo-blood group antigens for example are known modifiers of disease [16,17,18,19,20,21,22,23]. As originally proposed by Heim et al. [26], we believe the highly prevalent glycoproteins act as low-affinity binding site receptors for CTx influencing toxin action by enabling the initial binding of toxin to the intestinal cell surface. This precedes and likely enables toxin binding to the much more sparsely prevalent membrane glycosphingolipids, which act as the functional trafficking receptors enabling toxicity.

## 7. Take-Homes and Open Questions

In this review, we highlighted recent advances in our understanding of membrane biology and biophysics obtained through the use of CTxB. One important conclusion that emerges from these studies is that CTxB is not simply a passive reporter. It can drive phase separation, induce membrane curvature, stabilize rafts, stimulate its own internalization into cells, and re-direct GM1 into different intracellular trafficking pathways. This is not a new message, but it is one worth repeating given CTxB is still sometimes assumed to represent a benign raft and endocytic marker in the literature.

It is thus essential to exercise caution when using CTxB as a probe for membrane organization and trafficking mechanisms, especially in poorly defined systems.

It is also becoming increasingly clear that not all GM1s are created equal. Depending on its acyl chain structure and degree of saturation, GM1 can associate with ordered or disordered membrane phases in model systems, and form nanoclusters- or not- in cell membranes. CTxB itself also behaves very differently depending on whether it is bound to one or more copies of GM1, as well as their structural features. This can have critical consequences for CTxB’s ability to associate with rafts, bend membranes, and trigger endocytosis. Even changing the GM1/ CTxB ratio is sufficient to evoke some of these changes. Finally, it is important to recognize that lower affinity receptors for CTxB also exist, including other glycolipids and fucosylated secondary receptors. The membrane remodeling activities of CTxB are thus highly context-dependent.

The recent pandemic has highlighted the importance of understanding the varied mechanisms by which pathogens gain entry into cells. Many of the membrane remodeling activites of CTxB are shared with other members of the AB_5_ toxin family and some viruses [68,76,183]. For example, binding of certain viruses to cells via glycolipid receptors is thought to trigger similar mechanisms that facilitate their endocytic uptake [68,183]. The GL-Lect hypothesis suggests an intriguing mechanism by which this might occur [77,116,118,146,184,185]. Understanding how these events are orchestrated could provide essential insights into how the uptake of multiple classes of pathogens could be blocked- or how the internalization of specific pathogens could be inhibited while leaving endogenous endocytic pathways intact. Finally, the pathways uncovered through studies of CTxB hold the potential to be targeted for the delivery of large drug molecules [161]. Thus, the tricks developed by CTxB to enable CTx to enter cells may ultimately be exploited for pharmacological purposes.

## Data Availability

Not applicable.
